# Clinical diagnosis and management strategies for Macrodystrophia Lipomatosa: Insights from a rare case report

**DOI:** 10.1016/j.ijscr.2025.111751

**Published:** 2025-08-05

**Authors:** Yuni Artha Prabowo Putro, Dhandia Rifardi, Ery Kus Dwianingsih, Amri Wicaksono Pribadi, Rahadyan Magetsari, I. Made Dolly

**Affiliations:** a Department of Orthopedics and Traumatology, RSUP Dr. Sardjito Hospital, Jl. Kesehatan Sendowo No.1, Sleman, 55281, D.I.Yogyakarta, Indonesia; b Faculty of Medicine, Public Health and Nursing, Universitas Gadjah Mada, Jl. Farmako, Sendowo, Sekip Utara, Sleman, 55281, D.I.Yogyakarta, Indonesia; c Department of Anatomical Pathology, Faculty of Medicine, Public Health and Nursing, Universitas Gadjah Mada, Dr Sardjito General Hospital, Jl. Farmako, Sendowo, Sekip Utara, Sleman, 55281, D.I.Yogyakarta, Indonesia; d Department of Radiology, Faculty of Medicine, Public Health and Nursing, Universitas Gadjah Mada, Jl. Farmako, Sendowo, Sekip Utara, Sleman, 55281, D.I.Yogyakarta, Indonesia

**Keywords:** Macrodactyly lipomatous, Clinicopathological conference, Ray amputation, Functional outcome, Case report

## Abstract

**Introduction:**

Macrodystrophia lipomatosa (MDL) is a rare, congenital, non-hereditary disorder characterized by localized gigantism of the fingers or toes, resulting from the overgrowth of mesenchymal tissues, particularly fibroadipose tissue. The pathophysiology remains debated, with theories involving humoral, vascular, neurological mechanisms, and recent studies suggesting potential links to the PIK3CA gene. MDL lacks established diagnostic criteria and management guidelines, with treatment strategies ranging from conservative monitoring to surgical intervention.

**Case presentation:**

A 31-year-old female with a history of progressive enlargement of the right second toe, present since age 2, was referred for chronic pain and functional impairments. Imaging revealed multiple exostoses and soft tissue swelling. Core and open biopsies confirmed the diagnosis of MDL, showing adipose and connective tissue. Based on these findings, a second ray amputation was performed. Postoperatively, the patient showed significant improvement, with complete wound healing and a reduction in pain and functional limitations.

**Discussion:**

MDL is most commonly observed in males and affects the hands and feet, with unilateral involvement often seen. Imaging modalities play a crucial role in diagnosis. Histopathological examination reveals excessive adipose tissue. The progressive nature of MDL often leads to recurrence, requiring repeated surgical interventions. The second ray amputation performed in this case resulted in improved function and pain relief, as evidenced by favorable VAS and FAOS scores.

**Conclusion:**

This case highlights the importance of early diagnosis and individualized treatment for MDL. While surgical intervention can improve outcomes, further research is needed to establish standardized management protocols for this rare condition.

## Highlights

• Macrodystrophia lipomatosa (MDL) is a rare condition characterized by localized gigantism of fingers or toes due to fibroadipose tissue overgrowth.
• MDL lacks clear diagnostic criteria and management guidelines, making diagnosis and treatment challenging
• Personalized treatment and early surgical intervention can improve outcomes, but standardized guidelines for MDL management are still needed.
• This case emphasizes the importance of clinicopathological conferences for rare conditions like MDL, aiding in accurate diagnosis and treatment planning.
• Based of this findings, further research is needed to establish standardized management protocols for this rare condition.

## 1 Introduction

A rare, congenital, non-hereditary disease called macrodystrophia lipomatous (MDL) is characterized by localized gigantism of the fingers or toes. The condition is characterized by increased proliferation of mesenchymal components, including bone marrow, muscle, periosteum, and nerve sheaths, predominantly the fibroadipose tissue [1]. The MDL was originally used by Feriz in 1925 [2]. This condition can cause pain, change the natural aesthetic shape of the foot, and lessen the usefulness of the lower extremities, all of which can have an impact on the everyday lives and emotional well-being of patients [3]. In the literature, less than 100 cases of MDL have been reported. There remains a significant lack of consensus regarding the diagnostic criteria and management strategies for this condition and must be differentiated from various other disorders as the disease course, prognosis, complications, and therapies vary [4].

Management strategies for MDL are inconsistent, with approaches ranging from conservative monitoring to surgical interventions. Surgical techniques have been reported with varying degrees of success, but there is no universally accepted guideline for when to intervene surgically [[5], [6], [7]]. The lack of consensus on treatment protocols is exacerbated by the rarity of the condition, which limits the accumulation of large-scale clinical data to inform best practices [8]. As a result, clinicians often rely on anecdotal evidence and case reports to guide their management decisions, leading to a fragmented understanding of effective treatment modalities [9,10].

This study highlights the importance of a multidisciplinary diagnostic approach in MDL, including clinical assessment, radiographic interpretation and histopathological correlation, which were essential in guiding the appropriate treatment strategy. The surgical decision-making process was carefully tailored to the patient's functional limitations and personal goals, emphasizing the role of individualized management. This report is presented in compliance with the SCARE 2025 guidelines [11].

## 2 Case of presentation

A 31-year-old female patient was referred due to enlargement of the right second toe and chronic pain (VAS 5 out of 10). The patient reported that the growth, present since the age of 2, was increasing in size relative to the other toes and causing discomfort. Her condition necessitated that she could not wear identical shoes on both feet, resulting in her walking barefoot and experiencing difficulty in ambulation. The patient took analgesics to alleviate the pain. There was no record of similar complaints among family members.

**Fig. 1:**
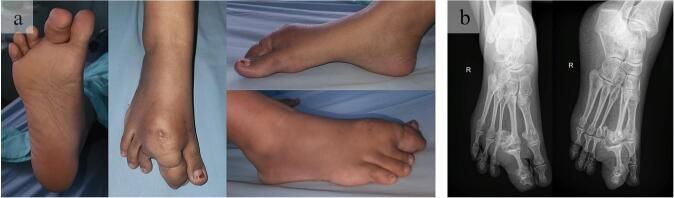
An initial (a) localized examination of the second toe of the right foot revealed a lump measuring 5x2x2 cm and (b) preoperative radiographic showing multiple exostosis on the metatarsal, proximal phalanx and distal phalanx.

The patient's vital signs were within normal limits. The right second toe appeared enlarged, measuring 5x2x2 cm, and was fixed and painful. The range of motion was limited due to pain, while sensory function was normal compared to the surrounding and contralateral sides. An X-ray of the right foot revealed multiple exostoses on the metatarsal, proximal phalanx, and distal phalanx, along with soft tissue swelling at the second toe **(**Fig. 1**).** To confirm the diagnosis, a clinicopathological conference was held, and a core biopsy was performed. Histological examination revealed adipose and connective tissue without tumor components, confirming the diagnosis of macrodystrophia lipomatosa. Based on these findings, the decision was made to proceed with a second ray amputation, as the surgical approach was deemed safe and appropriate.

The surgical decision based on clinical and patient functional consideration. The osseous hypertrophy with pain and joint instability affecting the feasibility if conservative debulking. The pain described by the patient significantly affecting the daily activity. Second-ray amputation offered better long term control of potential recurrence and maintaining functional of the foot.

Intraoperatively, the patient was positioned supine under general anesthesia, with a tourniquet applied to the right thigh for bleeding control. An inverted fishmouth-shaped incision was made on both the dorsal and plantar aspects, allowing for dissection in layers to expose the metatarsal bones.

**Fig. 2:**
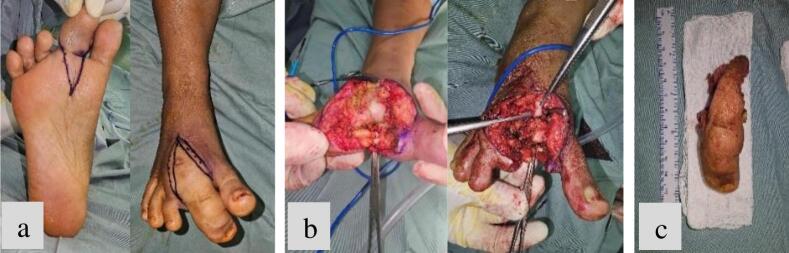
Clinical overview of intraoperative procedures ranging from (a) incision design, (b) second-ray amputation and (c) sample biopsy.

**Fig. 3:**
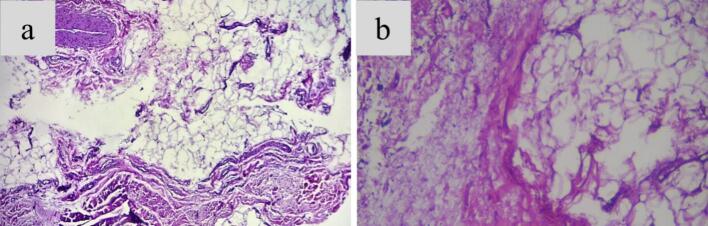
Histopathology of (a) core biopsy (b) open biopsy showed fat and connective tissue with extravasation of erythrocytes and the presence of lymphocytes, neutrophils, and macrophages.

An open biopsy of the amputated second toe **(**Fig. 2**)** was also performed and sent to the laboratory for analysis. Postoperatively, the gauze dressing was changed every two days, and the patient was given antibiotics for five days to prevent infection. A high-protein diet was recommended to support wound healing. One week after surgery, the results of the open biopsy confirmed the presence of mature fibroadipose tissue **(**Fig. 3**).**

**Fig. 4:**
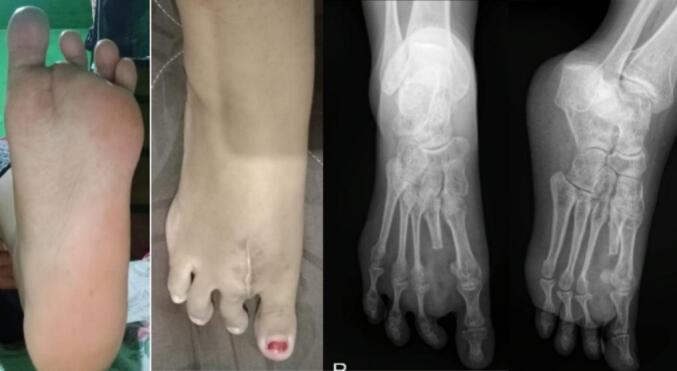
Follow up 4 months post surgery surgery. Postoperative x-rays after performing the second ray amputation showed no sign of recurrence.

**Fig. 5:**
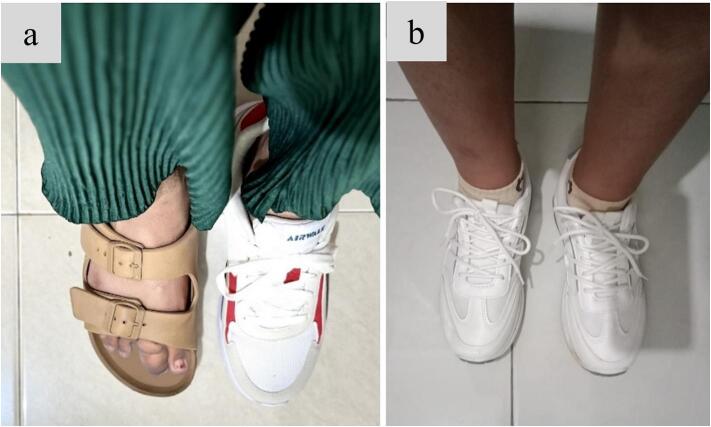
Clinical picture (a) The patient unable to wear same shoes on both feet. (b) Post-surgery: the patient able wear the same shoes on both feet without pain and walk longer without assistive devices (b).

**Fig. 6:**
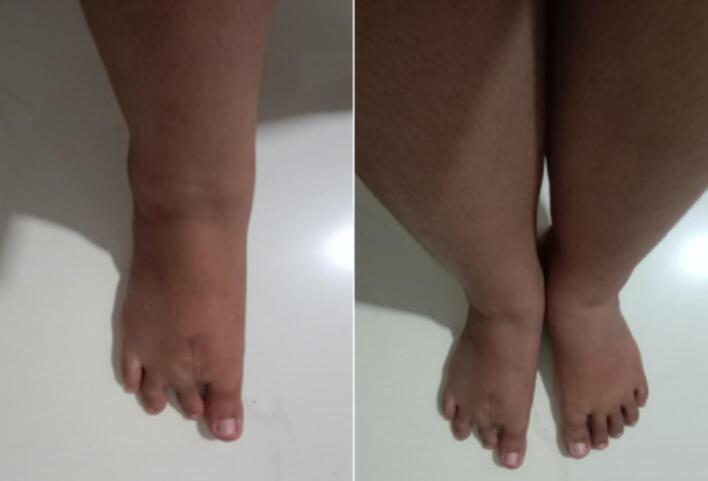
Follow up 11 months post surgery, no sign of recurrence. Gait showed balanced limb loading and no compensatory movements. Pain reduce and increase daily activity level.

Four months after surgery, the patient's wound had fully healed. A postoperative X-ray showed no signs of recurrence **(**Fig. 4**)**. The patient reported significant improvement, as she was able to perform daily activities comfortably, wear shoes without pain, and walk longer distances without the need for assistive devices **(**Fig. 5**)**. Follow-up assessment revealed positive outcomes, with the VAS score decreasing from 5 preoperatively to 1, and the FAOS score improving from 48 % to 92 %. The gait showed equal stride length, balanced limb loading and no compensatory movements. These improvements contributed to a better quality of life and enhanced functional capacity, marking key milestones in the patient's recovery. At 11 months post-surgery, the patient showed no signs of recurrence **(**Fig. 6**)**. Gait analysis revealed balanced limb loading with no compensatory movements. The patient reported a significant reduction in pain and an increase in daily activity levels.

## 3 Discussion

Macrodystrophia lipomatosa (MDL) is an uncommon, non-genetic congenital condition marked by localized gigantism resulting from the excessive proliferation of mesenchymal tissues, especially fibroadipose tissue [1]. The pathophysiology of MDL is contentious, encompassing ideas related to humoral, vascular, and neurological pathways, alongside current research suggesting possible links with the PIK3CA gene [11]. The PIK3CA gene encodes the catalytic subunit of PI3K, integral to the PI3K-AKT-mTOR pathway, which regulates cellular growth and metabolism. Mutations have been detected in affected tissues, resulting in mosaic pathway activation and localized tissue overgrowth including adipose, fibrous, and skeletal elements [12,13]. Histopathological and molecular analyses have consistently demonstrated these mutations in MDL affected digits, typical fibroadipose proliferation and adipose infiltration of nerve sheaths recognized as PIK3CA mutation driven anomalies [14]. These findings highlight the molecular basis also suggest PIK3CA mutation analysis can serve as a diagnostic tool in confirming macrodystrophia lipomatosa.

MDL is often categorised into progressive and static forms, with the progressive form being more prevalent [15]. In this case, the patient exhibited enlargement of the right second phalanges, evident for 2 years old. The patient had no familial history of similar problems and encountered considerable functional impairments, such as challenges in donning footwear and ambulating. Prasetyono and Hanafi's investigation of 108 MDL cases revealed that the hands and feet were the predominant locations, with unilateral distribution observed in the majority of instances [16]. The first, second, and third digits were predominantly engaged, either individually or collectively. Ray amputation is often preferred due to a significantly lower recurrence risk. Soft tissue debulking recurrence rates 33–60 % and may lead to complications such as scarring, joint stiffness, and impaired function [17]. In contrast, ray amputation marked functional improvement and significant decreases in foot width and area, further supporting the efficacy [18].

Imaging is essential for diagnosing MDL, as X-rays demonstrate distinctive hypertrophy of bone and soft tissue, occasionally resulting in a mushroom-like appearance. CT scans generally reveal excessive osseous growth and proliferation of adipose tissue, but MRI scans exhibit a high density of adipose tissue with a signal intensity akin to that of normal subcutaneous fat [1],[20] X-ray imaging disclosed several exostoses on the metatarsals and phalanges, corroborating the diagnosis. Histopathological analysis by core and open biopsies validated the existence of adipose and connective tissue, in accordance with established literature which shows an increase in adipose tissue scattered in fibrous tissue that can involve bone marrow, periosteum, muscle, nerve sheaths, and subcutaneous tissue [1], [20].

A clinicopathology conference was convened to deliberate on the diagnosis and treatment approach, consistent with the utilisation of such conferences as instructional instruments to examine rare cases from various viewpoints and achieve an agreement on therapy [19]. The rarity of MDL and the absence of established care protocols provide difficulties for clinicians. The progressive characteristics of MDL, along with a recurrence incidence of 33–60 % post-surgery, may necessitate repeated surgical procedures for conclusive treatment [17]. A second ray amputation was conducted to enhance functionality and alleviate discomfort, with the objective of providing the patient with pain-free, functioning foot capable of fitting into shoes and supporting a normal stride [4,18] Despite experiencing modest postoperative pain, the patient found it bearable, enabling her to walk greater distances, wear shoes, and regain independence. Subsequent evaluations with VAS and FAOS scores demonstrated substantial enhancement, with the FAOS score rising from 48 % to 92 %, and the VAS score declining from 5 to 1, indicating a favorable result. This case report contributes to the limited literature on MDL by clarifying the diagnostic approach and management strategies for this rare condition. The findings highlight the importance of personalized treatment and underscore the need for further research to develop more definitive guidelines for managing MDL. Limitation of this case is the short follow-up duration, limited the ability to assess long-term outcomes and potential recurrence.

## 4 Conclusion

This case highlights the importance of early diagnosis and individualized treatment for MDL. While surgical intervention can improve outcomes, further research is needed to establish standardized management protocols for this rare condition.

## Consent

Written informed consent was obtained from the patient for publication of this case report and any accompanying images.

## Ethical approval

This case report does not require ethical approval based on the research ethics committee's guidelines. It focuses on a patient's treatment and medical care, not research. Our institution's ethics committee confirmed that this report aligns with routine clinical practice and doesn't involve experimental interventions or additional data collection. We're ready to provide more information if needed, underscoring our commitment to ethical practices.

## Funding

This report received no specific grant from any funding agency in the public, commercial, or not-for-profit sectors.

## Author contributions

**Y.A. P. P** (Conceptualization, Writing- Original Draft, Validation, Investigation), **D.R:** (Validation, Methodology, Writing- Review & editing, Supervision) **E.K·D**: (Writing- Original, Draft, Resources, Validation), **A.W·P:** (Visualization, Resources, Methodology, Supervision) **A. F. H**: (Resources, Writing- Original Draft, Visualization, Validation), **R.M: (**Validation, Methodology, Writing- Review & editing, Supervision), **I.M.D:** (Resources, Writing- Original Draft, Visualization, Validation).

## Guarantor

Y.A.P·P.

## Research registration number

Not applicable

## Conflict of interest statement

The authors declare no conflicts of interest regarding the publication.

## Data Availability

Supporting data will be available upon reasonable request.
